# Natural Tendency towards Beauty in Humans: Evidence from Binocular Rivalry

**DOI:** 10.1371/journal.pone.0150147

**Published:** 2016-03-01

**Authors:** Ce Mo, Tiansheng Xia, Kaixin Qin, Lei Mo

**Affiliations:** 1 Center for Studies of Psychological Application, South China Normal University, Guangzhou, China; 2 Business School, Sun Yat-sen University, Guangzhou, China; University of Montreal, CANADA

## Abstract

Although human preference for beauty is common and compelling in daily life, it remains unknown whether such preference is essentially subserved by social cognitive demands or natural tendency towards beauty encoded in the human mind intrinsically. Here we demonstrate experimentally that humans automatically exhibit preference for visual and moral beauty without explicit cognitive efforts. Using a binocular rivalry paradigm, we identified enhanced gender-independent perceptual dominance for physically attractive persons, and the results suggested universal preference for visual beauty based on perceivable forms. Moreover, we also identified perceptual dominance enhancement for characters associated with virtuous descriptions after controlling for facial attractiveness and vigilance-related attention effects, which suggested a similar implicit preference for moral beauty conveyed in prosocial behaviours. Our findings show that behavioural preference for beauty is driven by an inherent natural tendency towards beauty in humans rather than explicit social cognitive processes.

## Introduction

A central challenge in behavioural psychology is to explore different aspects of human preference for their critical role in guiding and shaping individual behaviours. For the past decade, numerous studies have characterized preference for altruism, fairness, empathy, cooperation and prosociality along with the associated behavioural patterns in human adults and infants [1–8]. In contrast, however, much less is known about the preference for beauty that commonly occurs in every aspect of our daily lives from choosing our clothes to making new friends. According to Haidt & Keltner [9], the notion of beauty may refer to physical attractiveness expressed via sensory modalities, i.e. audiovisual beauty, or may reflect humaneness and virtue, which rely on the understanding of social rules that are independent of perceivable physical forms, i.e. moral beauty [10–13]. Audiovisual beauty refers to the sense of beauty that is directly based on the auditory pathway and/or visual pathway, which is familiar in individuals’ perceptual experience of the world and implies that sensory and symbolic elements of aesthetic objects conform to certain aesthetic rules (e.g. color, proportion and texture) [14, 15]. By contrast, moral beauty involves complex social meanings and refers to the expression of humanity, virtue, and talents which are independent of perceivable physical forms, based on the understanding of social rules and involve highly developed social affects and cognitions [10, 11, 15–17]. Traditionally, psychologists suggest the moral judgments are the results of reasoning and “higher” cognition [18]. However, Haidt et al. propose that human beings can determine moral judgment with intuition [19], and the difference between moral goodness and moral beauty relys on emotional response and motivation. In particular, Haidt [20] deems that charity, kindness, loyalty, self-sacrifice, courage and the other virtues are generally considered signs of moral goodness. An observer can cognitively identify an act as morally good but remain unmoved. However, when one similar act is considered to be moral beauty, it implies that the observer’s emotion has been moved by the act [9, 11, 16, 17].

A prominent theory in aesthetics proposes that our preference for beauty, given its spontaneous and compelling influence on our behaviour, is driven by a natural tendency encoded in our minds intrinsically rather than an explicit real-time social cognitive processing of the contextual information [14]. The natural tendency refers to an automatic influence driven by effortless perceptual activity towards the outside world, whereas social cognitive processing usually involves intentional coding, storing, retrieving and processing related information in specific social contexts [21]. Though it is largely based on theoretical speculation, there are some experimental researches trying to testify this conjecture. Some studies have shown that participants tended to look longer at attractive than unattractive faces [22–24]. Shimojo et al. [25] employed the gaze bias paradigm and found a gaze “cascade effect” when observers were instructed to decide which face in a pair of faces was more attractive, and the results suggested that preference for facial attractiveness is intrinsically linked in a positive feedback loop leading to conscious choice.

However, other studies have deemed that attractive faces can be perceived under constrained viewing conditions [22, 24]. One study revealed that facial attractiveness could be processed even when faces were only shown for 13 ms, a presentation time that is too short to accurately report seeing the faces [26]. Sui [27] further documented that compared to unattractive faces, the presence of task-irrelevant attractive faces significantly lengthened task performance in a spatial cuing task, which suggested that facial beauty automatically captured individuals’ attention. Another study also revealed that beauty was not only detectable at parafovea but also at periphery, which implied that the preference for facial attractiveness may not require voluntary effort [28]. In addition, neural activities related to the perception of attractive faces were engaged even when participants were not asked to provide explicit judgments of attractiveness [22]. Similarly, McDonald et al. [29] used skin conductance responses to reveal that physiological responses were detectable even without requiring explicit attractiveness evaluations,. These findings support that facial attractiveness is appraised rather automatically.

Similar to the argument on facial attractiveness, there are two views on moral judgment. The empiricist posits that moral knowledge, moral beliefs, and all the other stuff of morality can be learned in everyday life (as in Kohlberg’s post-conventional reasoning). However, the nativist deems that knowledge about such issues as fairness, harm, and respect for authority may be built into the human mind by evolution, therefore, most of the cognitive process of moral judgment can be referred to as an intuitive, or automatic system [19]. Recently, several empirical evidences supported nativist’s view [30]. Neuroimaging studies of moral judgment in normal adults emphasized the role of emotion on moral cognitive process[18], which suggested that the identifying of moral beauty may be automatic.

**Fig 1:**
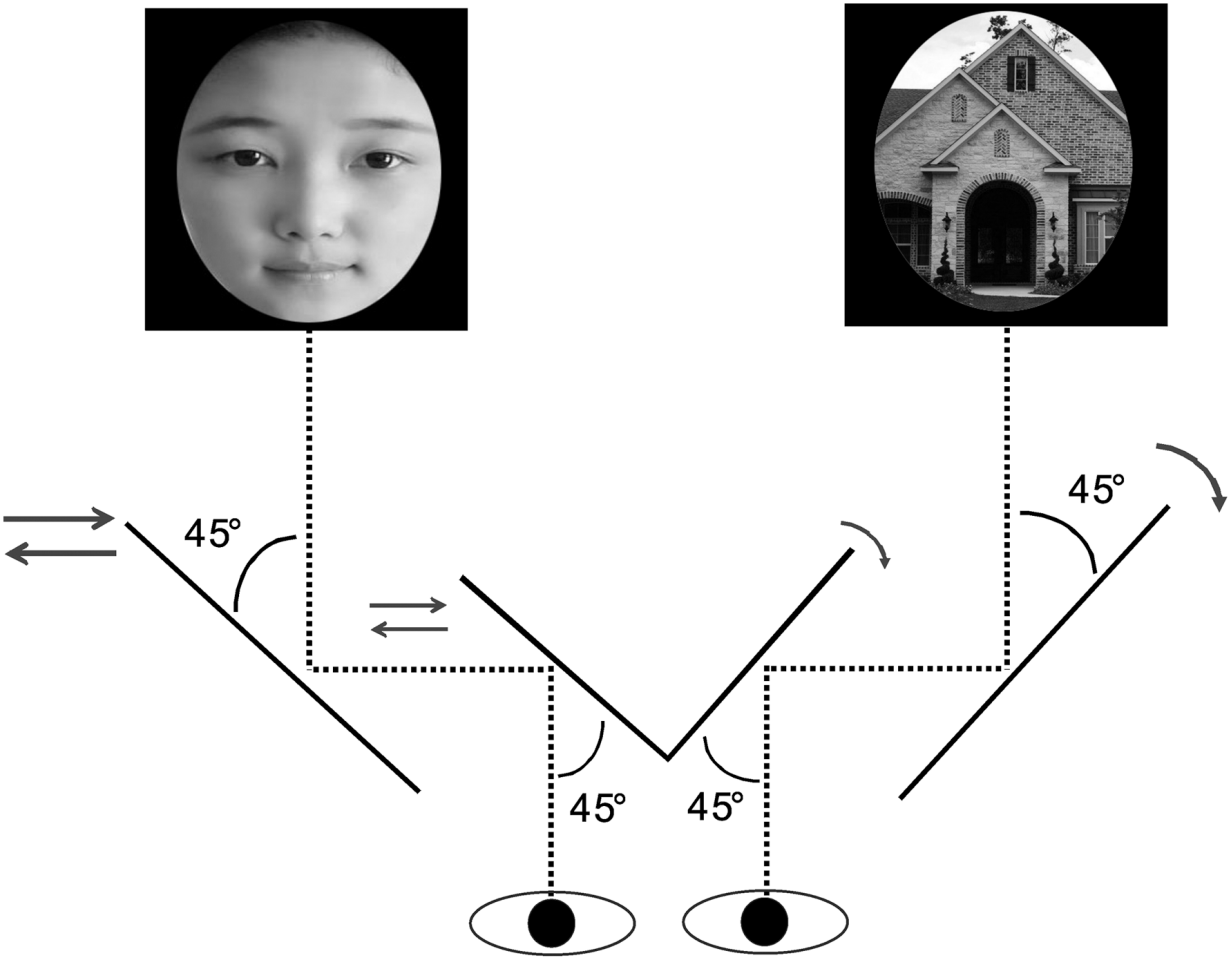
Illustration of binocular rivalry paradigm. Participants viewed two visually distinctive objects through a stereoscope that simultaneously projected the stimuli to different eyes’ fields, thus creating the binocular rivalry condition. Implicit perceptual selectivity to a stimulus can be measured by total length of time it dominates in visual awareness.

A convincing approach to gain further insights into the human preference for beauty is the binocular rivalry paradigm, a procedure with a well-known capacity for examining human subconscious selectivity, free from the confounding effects of explicit task requirements and cognitive processing (e.g. conscious information processing) [31, 32]. In this paradigm, two perceptually distinctive stimuli, e.g. a face paired with a house, are presented to different visual fields using a stereoscope. The unresolvable visual input dichotomy in turn leads to a phenomenon known as perceptual dominance competition between the two stimuli, in which each stimulus alternates between the status of being consciously perceived (perceptual dominance) and unseen (perceptual suppression) [33]. By measuring the overall length of time the target stimulus is selected for visual awareness (dominance duration), we are able to quantify the participant’s selectivity to the stimulus in question (see Fig 1). This technique has been employed in a number of studies comparing human subconscious selectivity to stimuli differing in physical properties [34–36] or overt affective values [37, 38].

In the current study, we used a binocular rivalry paradigm to systematically explore humans’ putative natural tendency toward beauty. We employed two kinds of beauty: visual beauty (e.g. facial attractiveness) and moral beauty (e.g. altruistic behaviour). If human beings’ preference for beauty is natural and without explicit effort, we would expect that under the absence of explicit tasks and other social cognitive demands, there are longer perceptual dominance duration on attractive faces than unattractive ones; furthermore, this preference would lead similar perceptual dominance enhancement for virtuous persons irrespective of the degree of their facial attractiveness. On the contrary, if the preference for beauty requires cognitive effort, under the absence of explicit tasks and other social cognitive demands, human beings would not show perceptual dominance duration for attractive faces or the persons with moral beauty compared with unattractive faces or the persons with moral neutrality.

To test these hypotheses, we conducted three experiments. The Experiment 1 sought to investigate the preference for visual beauty by comparing the subconscious selectivity to faces of different physical attractiveness levels (beautiful, average-looking, unattractive). Experiment 2 sought to investigate the preference for moral beauty by comparing the selectivity to average-looking characters associated with different moral affective values (virtuous, neutral, immoral) using the Affective Learning Procedure [38, 39]. Due to the vigilance-related attention bias induced by negative social information that has been reported [40, 41], Experiment 3 was conducted to further examine the influence of positive moral affective values in the absence of negative moral affective values. In all three experiments, participants were presented with photos of persons differing either in physical attractiveness or moral affective values. Each face was randomly paired with one of two pre-selected photos of a house. Participants were instructed to perform a binocular rivalry task on the simultaneously presented face and house which required them to indicate what they had visually perceived. Subconscious selectivity to given stimuli was measured as the total dominance duration during the binocular rivalry trial.

In addition, some studies have found that facial perception may be modulated by gender [22, 25, 42–44]. Ishai [45] found an interaction between the perceptions of facial attractiveness and the sexual preference of the participant: in heterosexual women and homosexual men, attractive male faces elicited stronger activation than attractive female faces, whereas in heterosexual men and homosexual women, attractive female faces evoked stronger activation than attractive male faces. Hence, gender was considered as a factor in the analyses in our experiments.

## Methods

### Ethics statement

**Fig 2:**
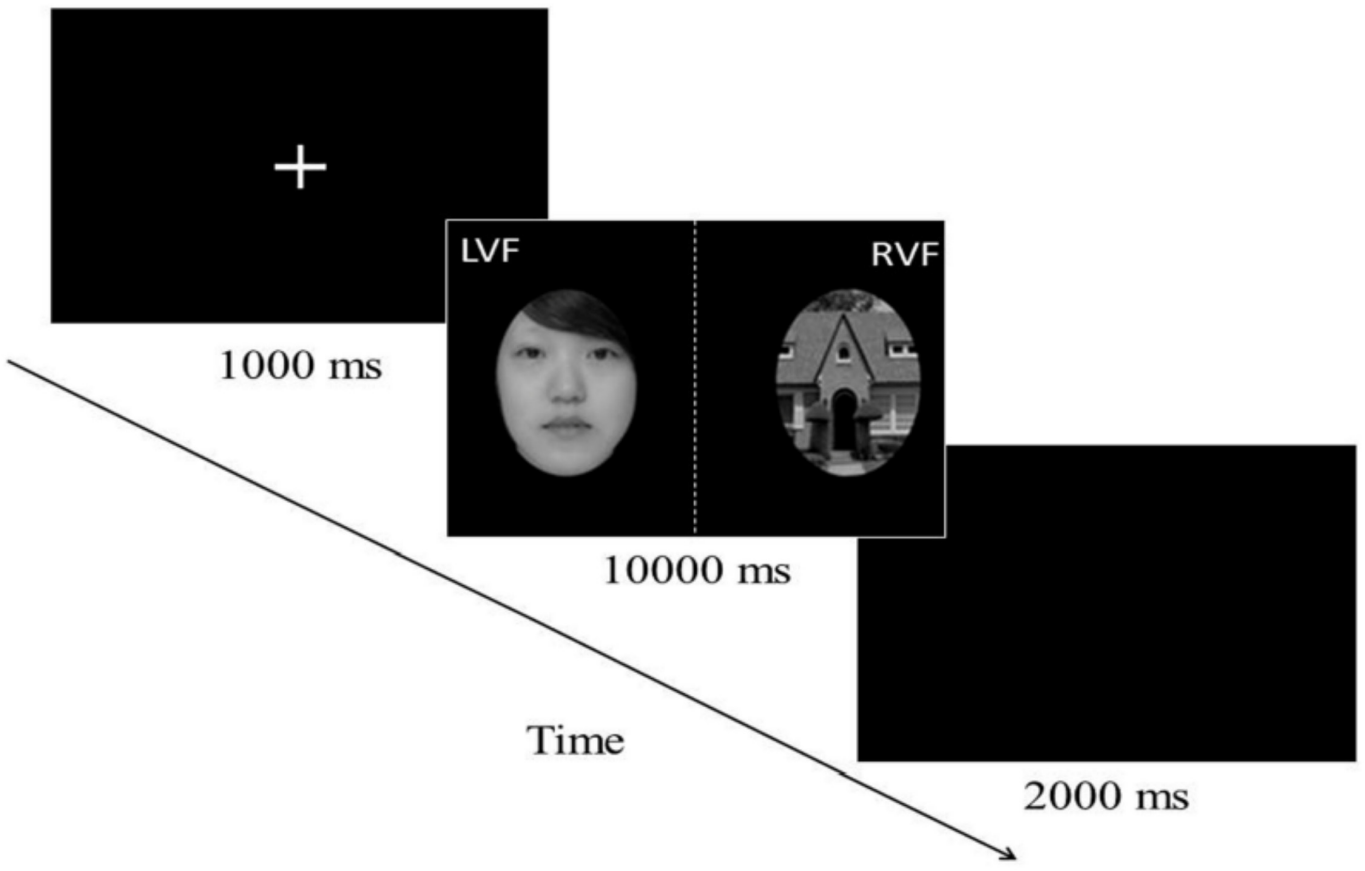
Binocular rivalry task trial procedure.

**Fig 3:**
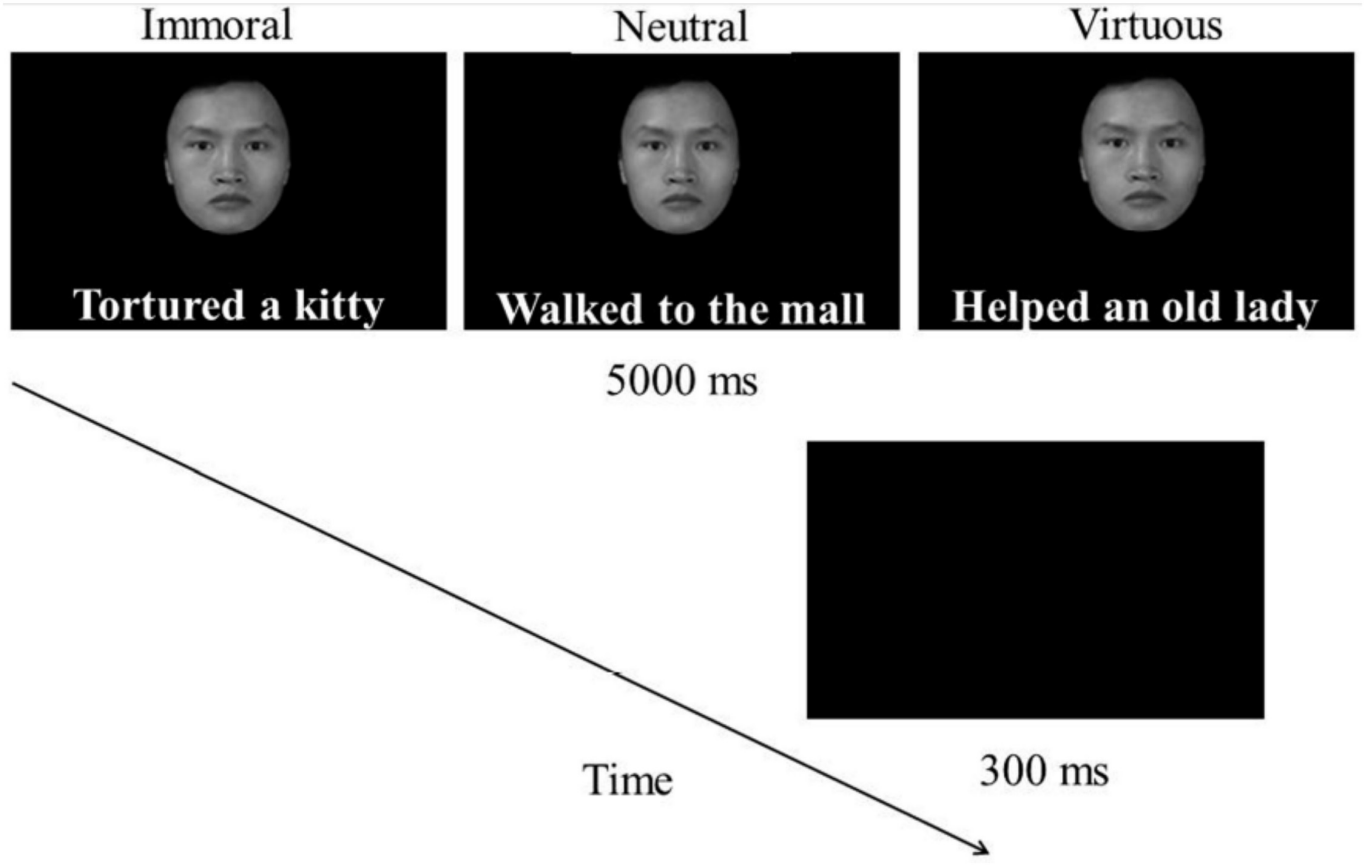
Illustration of stimuli types and trial procedure of affective learning.

The study protocol was approved by the South China Normal University Research Ethics Committee, and the research was conducted in accordance with the ethical standards specified in the 1964 Declaration of Helsinki and its later amendments. Written informed consent was obtained from the participants, and they could freely withdraw from the study at any time. The individual in this manuscript whose images were presented in Figs 1–3 have given written informed consent (as outlined in the PLOS consent form) to publish these case details.

### Participants

All participants were recruited from healthy undergraduate students at South China Normal University. They were aged 18 to 25 with normal or corrected-to-normal visual acuity. Students who wore glasses were excluded due to the incompatibility between their glasses and the stereoscope used for creating binocular rivalry. Prior to the experiment, participants, unaware of the scope and the objective of the current research project, were briefed on the experimental procedure and task instructions. Data from six participants (two in Experiment 1, four in Experiment 2) were excluded from statistical analyses due to noncompliance with the experimental protocol (e.g. they reported only blended percepts or pressed invalid keys in more than one third of the total trials).

A composite open adaptive stopping rule [46] was applied to determine the sample sizes. This method permits the researcher to perform a statistical test at any time. If the outcome of this statistical test is *p <* .01, then the researcher stops testing participants and rejects the null hypothesis; in contrast, if *p >*.36, then the researcher stops testing participants and does not reject the null hypothesis. If .01 < *p* < .36, then more participants are tested. In the current study, the initial target sample size was set as one third of the sample size reported in previous studies [37, 38]. Data were collected from the initial participant group and *p* values of the planned statistical analyses were checked to see if they fell between 0.01 and 0.36, in which case additional participants were included until *p* values were less than 0.01 [47]. It is important to note that the final number of participants in current study is fewer or equal to those reported in previous studies. After the experiment, all participants received a small monetary reward for participation.

### Materials

Two types of experimental stimuli were involved in the current study: photos of faces and behaviour descriptions. Physical attractiveness of faces in the photos and the moral affective value of behavioural descriptions were assessed in a pilot study by independent groups of participants who were not involved in the current experiments. Based on previous studies [9, 48], moral beauty refers to an act as moral goodness and makes the observers moved (e.g. eliciting the moral emotion of elevation). Thus, we asked participants to assess the moral affective value of behavioural descriptions, and if a behaviour is assessed to elicit more positive moral emotion, it would be identified to be closer to moral beauty, whereas if a behaviour is assessed to elicit negative moral emotion, it would be identified to be closer to immoral states. For the facial stimuli selection, 100 candidate gray-scale photos of human faces were preprocessed to share the same size and resolution (70 × 95 pixels) using Photoshop CS 4.0 (Adobe Systems Incorporated). The luminance was measured using a light meter and the contrast was calculated as the mean luminance difference between the stimulus region (face and house) and back ground region which was then divided by the average luminance of the photos. An oval window was superimposed on the centre of each photo to mask out the hair, accessories and neck. A total of 57 participants were recruited to rate the candidate face stimuli according to degree of physical attractiveness on a 7-point scale (1 for unattractive, 7 for beautiful). According to their attractiveness ratings, 24 photos were selected and categorised into three non-overlapping, equal-sized sets (mean ratings: unattractive = 2.03, average-looking = 4.07, beautiful = 6.12). Within each set, half of the faces were female. To create the binocular rivalry condition, each of the selected faces was paired with one of two novel gray-scale photos of houses which went through the same image processing steps described above. Visual features of no interest were held constant across all stimuli to control for confounding effects caused by irrelevant physical variations.

Similarly, a total of 48 candidate behaviour descriptions were assessed for morality affective values by another 34 participants on a 7-point scale (1 for immoral, 7 for virtuous). According to their morality ratings, 18 behavioural descriptions were selected and categorised into 3 equal-sized sets (mean ratings: virtuous = 5.53, neutral = 4.17, immoral = 2.12). To statistically validate the cross-set heterogeneity, two repeated measures ANOVAs were conducted on the physical attractiveness ratings and morality affective value ratings. As expected, we found a significant difference across the three photo sets and the three description sets (facial stimuli: *F* = 268.7, *P*< 0.001; Post-hoc Analyses: beautiful > bverage-looking > unattractive. Behaviour Descriptions: *F* = 685.52, *P* < 0.001; Post-hoc Analyses: virtuous > neutral > immoral; *P* < 0.05 corrected for multiple comparisons), indicating that our material selection was in agreement with our experimental design.

### Experimental Procedure

All instructions and stimuli were presented using E-Prime2.0 (Microsoft Inc.) on a 17-inch LCD flat-screen monitor. Behavioural responses were collected using a computer keyboard of standard layout. The experimental procedure involved two independent sessions. The *Binocular Rivalry Task Session* measured participants’ facial selectivity, and the *Affective Learning Session* involved learning the correspondence between character faces and their moral affective values. The Binocular Rivalry Task procedure was included in all three experiments, whereas the Affective Learning procedure was only included in Experiment 2 and Experiment 3.

In Experiment 1, 29 participants (15 males) completed the Binocular Rivalry Task Session. For this task, they were seated with their heads fixed in a comfortable position and viewed stimuli through a mirror stereoscope at a distance of approximately 55 cm. They were presented with the face photos whose physical attractiveness had been assessed as described previously. At the beginning of each trial, a fixation cross was presented for 1 s. Subsequently, the face-house photo pair was displayed for 10 s. Consecutive trials were separated by a blank screen for 2 s (see Fig 2). Each face-house pair was presented four times in a counterbalanced order. Participants were then instructed to press the corresponding button to indicate what they visually perceived (i.e. to press “A” for a face, “L” for a house and Space Bar for both). The visual field in which the facial stimulus was presented was counterbalanced across trials (half in the right visual field and half in the left) to eliminate perceptual laterality confounds. Dominance duration was recorded as the dependent variable in a total of 96 trials for each participant.

In Experiment 2, 31 participants (12 males) were presented with face photos of average-looking characters. Prior to the *Binocular Rivalry Task Session*, these photos were associated with behaviour descriptions of different moral affective values in the *Affective Learning Session*. In this procedure, participants viewed each face and its corresponding behaviour description presented simultaneously in pairs, with the facial stimulus above the description sentence. Average-looking faces (half female, mean rating = 4.07) were used in order to reduce undesired influence of variation in physical attractiveness across the facial stimuli. The face-sentence pair was displayed for 5s with a 300ms blank interval between trials (see Fig 3). Each pair was presented four times, during which participants were required to imagine the currently viewed person performing the behaviour described in the corresponding sentence and to make explicit moral judgments about the behaviour. They were instructed to press “A” for a virtuous act (e.g. helped an old lady cross the road), “L” for an immoral act (e.g. tortured a cat) and Space Bar for a neutral act (e.g. walking in the street). This moral judgment task was designed to reinforce the acquired connection between the character and their moral affective value. To further control for possible physical disparities in the stimuli, we created three counterbalanced versions of the task so that each face was associated with each moral affective value once. Each participant was assigned one version of the task. After learning the character-behaviour pairs, participants were given a memory test in which they were presented with the face photos they viewed earlier and were asked to recall and report the behaviour description associated with each character. Participants who failed to demonstrate 80% or above accuracy were required to repeat the learning process until this criterion was met. Once they achieved at least 80% accuracy, they proceeded to the binocular rivalry task session described previously. As in Experiment 1, dominance duration was recorded as the dependent variable in a total of 72 trials for each participant.

In Experiment 3, 31 participants (12 males) went through the same Affective Learning Session and Binocular Rivalry Task Session as in Experiment 2. We used the same stimuli and learning criterion as in Experiment 2. However, because we only investigated the effect of positive moral affective values in comparison to neutral values, we excluded the six descriptions of immoral behaviours and their corresponding characters. Because of this, only two counterbalanced task versions were created. Data were collected from a total of 48 binocular rivalry trials for each participant.

Following Anderson et al.[38], for each individual participant in all three experiments, we calculated the mean duration during which structurally neutral faces (previously paired unattractive, average-looking or beautiful faces in Experiment 1; paired virtuous, neutral or immoral behavioural descriptions in Experiment 2 and 3) were seen across trials (mean face dominance duration). We also computed a mean house duration reflecting the time during which a house was seen (this is equivalent to the index of mean face suppression duration when blended percepts are removed). We excluded data based on three criteria. First, we excluded outliers from the dominance duration that were outside the range of three standard deviations from the mean of each condition. Second, percepts occurring at the end of each trial were excluded from the dominance duration because they were artificially shortened by the end of the trial. Third, very brief percepts (less than 100ms) were excluded from all analyses because we took them to reflect slight differences in reaction time for pressing or releasing both keys to report blended percepts. Effect sizes for omnibus F-tests and planned post-hoc contrasts were estimated using partial *η*^2^ and Cohen’s *d* respectively.

## Results

**Fig 4:**
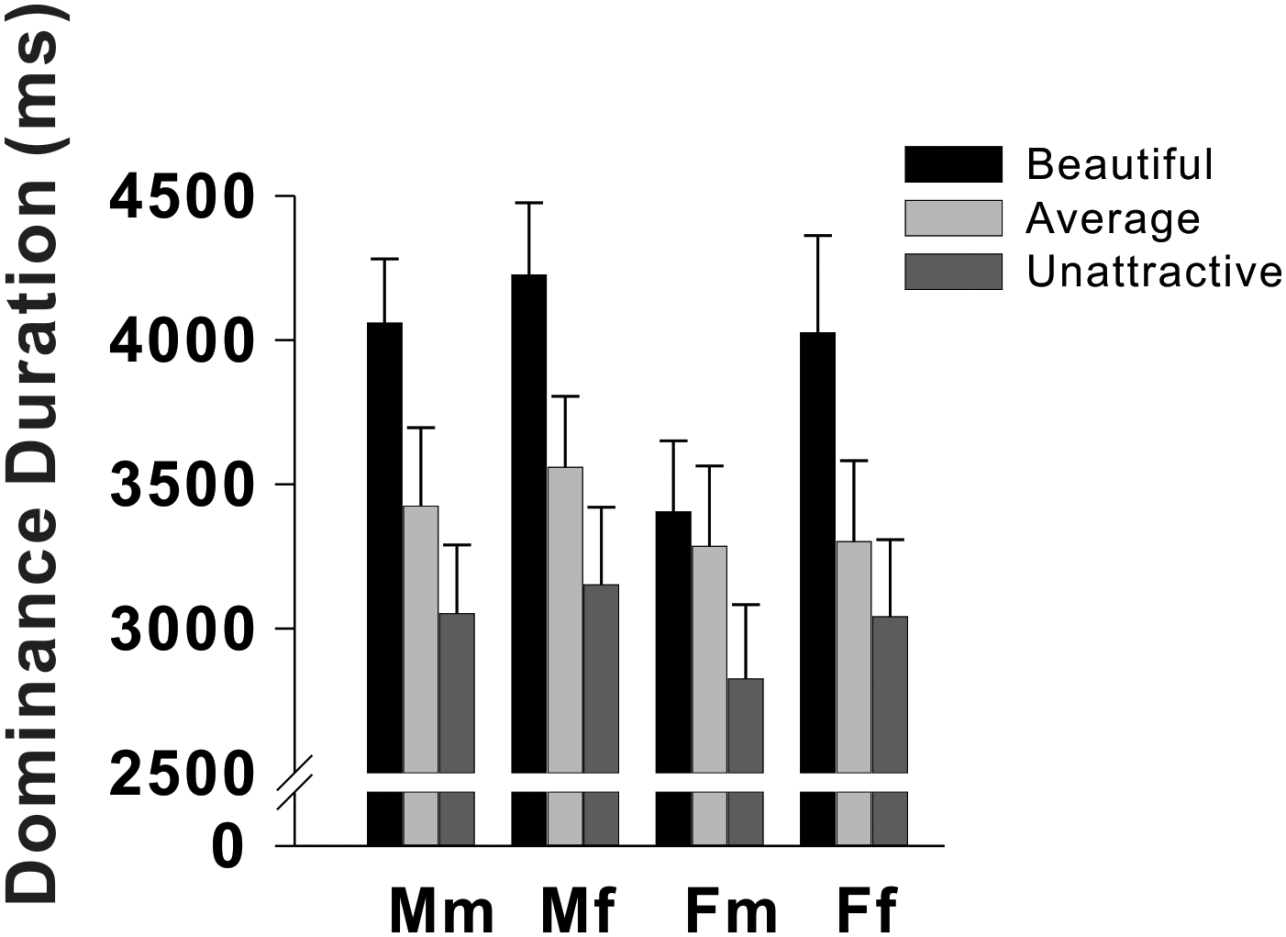
Dominance duration in Experiment 1. Mm = male participants presented with male faces. Mf = male participants presented with female faces. Fm = female participants presented with male faces. Ff = female participants presented with female faces.

For data analysis of Experiment 1, a 2 × 2 × 3 mixed-design ANOVA was conducted with participant gender (male, female) as the group factor and face gender (male, female) and physical attractiveness (unattractive, average-looking, beautiful) as the within-participant factors. We observed a significant difference in dominance duration across the three physical attractiveness levels (*F*_2,54_ = 26.81, *P* < .001, *η*^2^ = .498). Post-hoc pairwise comparisons further validated that dominance duration increased monotonically with facial beauty degrees (mean dominance duration: beautiful (3928ms) > average-looking (3392ms), *d* = 0.3813; average-looking (3392ms) > unattractive (3017ms), *d* = 0.3895; *P* < .05 corrected for multiple comparisons; see Fig 4 and Table A in S1 Text. Dominance duration was significantly longer for female faces (*F*_1,27_ = 7.143, *P* < .05, *η*^2^ = .228), but did not differ statistically between male and female participants(*F*_1,27_ = 0.282, *P* = .60, *η*^2^ = .01).

Importantly, we found no significant two-way interactions (participant gender × face gender: *F*_1,27_ = 1.03, *P* = .319,*η*^2^ = .037; physical attractiveness × face gender: *F*_2,54_ = 0.65, *P* = .529, *η*^2^ = .023; physical attractiveness × participant gender: *F*_2,54_ = 1.84, *P* = .169, *η*^2^ = .064) or three-way interaction (*F*_2,54_ = 1.383, *P* = .260,*η*^2^ = .049). This indicated that the dependency of visual dominance superiority on physical attractiveness was not modulated by the gender of the participants or faces. Rather, these results revealed subconscious selectivity for attractive faces regardless of gender difference, even when no explicit cognitive task requirements were involved. This suggests a preference for facial beauty that occurs naturally and effortlessly in humans.

**Fig 5:**
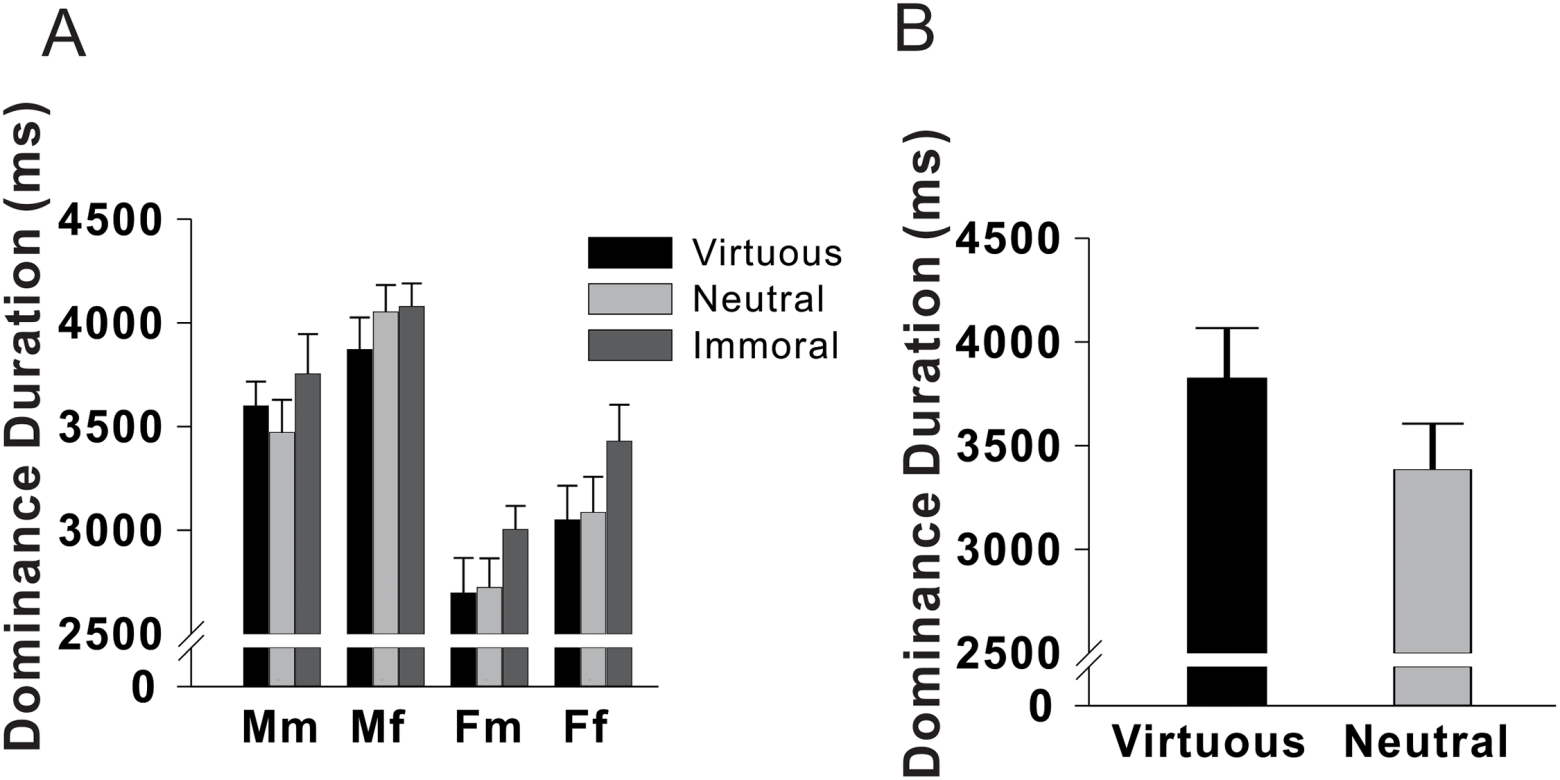
Dominance duration in Experiment 2 and 3. *A*: Experiment 2 results. *B*: Experiment 3 results. Mm = male participants presented with male faces. Mf = male participants presented with female faces. Fm = female participants presented with male faces. Ff = female participants presented with female faces.

Despite the enhanced perceptual dominance of attractive faces demonstrated in Experiment 1, it remained uncertain whether the implicit preference for beauty is restricted to physical forms. Hence, we conducted Experiment 2 to investigate whether human selectivity in the context of moral beauty would mirror the case of facial beauty. Data were analysed using a similar 2 × 2 × 3 mixed-design ANOVA as in Experiment 1, with the critical within-participant factor changed from physical attractiveness level to moral affective values (virtuous, neutral, immoral). We found a significant main effect of moral affective value (*F*_2,58_ = 3.42, *P* = .039, *η*^2^ = .106), which did not interact with participant gender or face gender (participant gender × face gender: *F*_1,29_ = 0.004, *P* = .95, *η*^2^< .001; moral affective value × face gender: *F*_2,58_ = 0.332, *P* = .719,*η*^2^ = .011; moral affective value × participant gender: *F*_2,58_ = 0.353, *P* = .704, *η*^2^ = .012). Interestingly, however, post-hoc analysis showed that dominance duration pertaining to immoral characters was significantly longer than that pertaining to virtuous and neutral characters, which did not differ significantly from each other (mean dominance duration: immoral (3566ms) > neutral (3333ms), *d* = 0.251; immoral (3566ms) > virtuous (3304ms), *d* = 0.247; *P*< .05 corrected for multiple comparisons; see Fig 5A and Table B in S1 Text). Additionally, we found significant main effects of face gender (*F*_1,29_ = 12.633, *P* = .001, partial *η*^2^ = .303) and of participant gender (*F*_1,29_ = 11.137, *P* < .005, partial *η*^2^ = .277).

The results of Experiment 2 were in agreement with previous findings that humans automatically prioritize their attention toward stimuli with socially negative or hostile implications as an instinctive countermeasure to protect themselves from potential danger [37–40], leading to longer dominance duration for characters associated with descriptions of immoral behaviours (e.g. theft, lying, violence). Hence, one possible explanation for the prolonged dominance of immoral characters in visual awareness is that it is caused by the increased vigilance level in response to potential threats, whose influence on human behaviour overwhelmed all intrinsic natural tendencies that play a lesser role in self-protection. However, it should be noted that such vigilance-related attention bias was unlikely to be relevant for tendency towards beauty per se; it may instead reflect the instinct of self-preservation common in all species, which stems from the long-term evolutionary process.

To test this possibility, we conducted Experiment 3 to examine whether participants would exhibit an implicit preference for virtuous characters that were not potential threats to security. As a result, the mean dominance duration for virtuous characters (3825ms) was significantly longer than that for neutral characters (3384ms) (paired-sample *t* = 3.749, *P* = .001, *d* = .493; see Fig 5B and Table C in S1 Text). Taken collectively, the results of Experiments 2 and 3 expanded previous findings [38] by revealing a vigilance-irrelevant preference for virtuous characters in addition to vigilance-induced attention to immoral characters. These findings suggest that the natural tendency toward beauty is not restricted to physical attractiveness, but also extends to positive social implications when an individual’s sense of security is not compromised.

## Discussion

The current research focuses on whether humans automatically exhibit preference for visual and moral beauty without explicit cognitive efforts. Across three experiments, we investigated the human preference for beauty. In Experiment 1, the significant main effect in dominance duration across three physical attractiveness levels and the post-hoc test (i.e. beautiful > average-looking > unattractive) indicated a natural tendency toward visual beauty. In the present study, we were not able to test what caused the attractiveness-dominance effect; however, previous studies have documented several factors that might influence the perception of physical attractiveness. These factors include facial characteristics (e.g. symmetry, sexually dimorphic shape cues, averageness, skin color/texture and cues to personality) [49–53], and several important sources of individual differences in face preferences (e.g. hormone levels and fertility, own attractiveness and personality, visual experience, familiarity and imprinting, social learning) [51, 54, 55]. These studies suggest that there is something universal about attractive faces (and unattractive faces) that is recognized both across individuals and cultures [27, 51, 56]. Subsequently, combining Experiments 2 and 3, we disentangled the implicit preference for beauty from explicit social cognitive contributions using a binocular rivalry paradigm. In addition to preference for physically attractive characters, we identified preference for virtuous persons when participants were not threatened by the presence of hostile and potentially dangerous characters. Together, our findings provide novel and compelling evidence in revealing a universal natural tendency towards both facial and moral beauty that is unrelated to contextual demands and other cognitive factors (e.g. moral beliefs, or moral reasoning).

Notably, our findings present a new challenge to the well-established theory of affective prejudice in humans, which claims that our impression of other people's character is biased by their physical appearance [57–61]. This idea is not supported by our experimental results because the vigilance-related dominance duration enhancement was observed only for characters paired with negative descriptions, indicating that it was the villainous characters rather than the unattractive ones that were intuitively identified as potential sources of danger. Hence, the physical attractiveness stereotype might reflect cognitive prejudice possibly derived from social interactions, which prevails only on the conscious level, whereas subconsciously physical appearance has little influence on our impression of other people’s characters.

Finally, our findings raise the possibility that the natural tendencies towards facial and moral beauty are associated with different functional characteristics respectively. It is likely that the former mainly involves sensory pleasantness while the latter primarily relies on internalized social rules, as suggested by the dissociation of the implicit behavioural patterns pertaining to unattractive and immoral characters. Future work is thus needed to further explore the functional properties of the human natural tendency towards beauty along with its cultural implications. In addition, the results in the present study cannot explain the interplay of moral beauty and physical attractiveness. We separately investigated the effect of visual beauty and moral beauty, with the results suggesting a natural tendency towards facial and moral beauty. However, there is likely to be an interaction between facial and moral beauty, and we will investigate it in further research.

## Supporting Information

S1 Text Materials, Methods and results in Experiment 1–3. (DOCX)
