# Supplementary material for: Natural Tendency towards Beauty in Humans: Evidence from Binocular Rivalry
Source: PLoS One. 2016 Mar 1;11(3):e0150147. doi: 10.1371/journal.pone.0150147 (PMC4773156; doi:10.1371/journal.pone.0150147)
Supplement: S1 Text — (DOCX) [file pone.0150147.s001.docx]

Supplementary Material

The supplementary material includes:

SOM text

Tables A-C

Materials and Methods.

*Participants.*

All participants were recruited from healthy undergraduate students at South China Normal University. They were aged 18 to 25 with normal or corrected-to-normal visual acuity. Students who wore glasses were excluded due to the incompatibility between their glasses and the stereoscope used for creating binocular rivalry. Prior to the experiment, participants, unaware of the scope and the objective of the current research project, were briefed on the experimental procedure and task instructions. Data from six participants (two in Experiment 1, four in Experiment 2) were excluded from statistical analyses due to noncompliance with the experimental protocol (e.g. they reported only blended percepts or pressed invalid keys in more than one third of the total trials).

*Stimuli Preparation and Assessment.*

Two types of experimental stimuli were involved in the current study: photos of faces and behaviour descriptions. Physical attractiveness of faces in the photos and the moral affective value of behavioural descriptions were assessed in a pilot study by independent groups of participants who were not involved in the current experiments. For the facial stimuli selection, 100 candidate gray-scale photos of human faces were preprocessed to share the same size and resolution (70 × 95 pixels) using Photoshop CS 4.0 (Adobe Systems Incorporated). The luminance was measured using a light meter and the contrast was calculated as the mean luminance difference between the stimulus region (face and house) and back ground region which was then divided by the average luminance of the photos. An oval window was superimposed on the centre of each photo to mask out the hair, accessories and neck. A total of 57 participants were recruited to rate the candidate face stimuli according to degree of physical attractiveness on a 7-point scale (1 for unattractive, 7 for beautiful). According to their attractiveness ratings, 24 photos were selected and categorised into three non-overlapping, equal-sized sets (mean ratings: unattractive = 2.03, average-looking = 4.07, beautiful = 6.12). Within each set, half of the faces were female. To create the binocular rivalry condition, each of the selected faces was paired with one of two novel gray-scale photos of houses which went through the same image processing steps described above. Visual features of no interest were held constant across all stimuli to control for confounding effects caused by irrelevant physical variations.

Similarly, a total of 48 candidate behaviour descriptions were assessed for morality affective values by another 34 participants on a 7-point scale (1 for immoral, 7 for virtuous). According to their morality ratings, 18 behavioural descriptions were selected and categorised into 3 equal-sized sets (mean ratings: virtuous = 5.53, neutral = 4.17, immoral = 2.12). To statistically validate the cross-set heterogeneity, two repeated measures ANOVAs were conducted on the physical attractiveness ratings and morality affective value ratings. As expected, we found a significant difference across the three photo sets and the three description sets (facial stimuli: F = 268.7, P< 0.001; Post-hoc Analyses: beautiful > bverage-looking > unattractive. Behaviour Descriptions: F = 685.52, P < 0.001; Post-hoc Analyses: virtuous > neutral > immoral; P < 0.05 corrected for multiple comparisons), indicating that our material selection was in agreement with our experimental design.

*Experimental Procedure.*

All instructions and stimuli were presented using E-Prime2.0 (Microsoft Inc.) on a 17-inch LCD flat-screen monitor. Behavioural responses were collected using a computer keyboard of standard layout. The experimental procedure involved two independent sessions. The Binocular Rivalry Task Session measured participants’ facial selectivity, and the Affective Learning Session involved learning the correspondence between character faces and their moral affective values. The Binocular Rivalry Task procedure was included in all three experiments, whereas the Affective Learning procedure was only included in Experiment 2 and Experiment 3.

In Experiment 1, 29 participants (15 males) completed the Binocular Rivalry Task Session. For this task, they were seated with their heads fixed in a comfortable position and viewed stimuli through a mirror stereoscope at a distance of approximately 55 cm. They were presented with the face photos whose physical attractiveness had been assessed as described previously. At the beginning of each trial, a fixation cross was presented for 1 s. Subsequently, the face-house photo pair was displayed for 10 s. Consecutive trials were separated by a blank screen for 2 s. Each face-house pair was presented four times in a counterbalanced order. Participants were then instructed to press the corresponding button to indicate what they visually perceived (i.e. to press “A” for a face, “L” for a house and Space Bar for both). The visual field in which the facial stimulus was presented was counterbalanced across trials (half in the right visual field and half in the left) to eliminate perceptual laterality confounds. Dominance duration was recorded as the dependent variable in a total of 96 trials for each participant.

In Experiment 2, 31 participants (12 males) were presented with face photos of average-looking characters. Prior to the Binocular Rivalry Task Session, these photos were associated with behaviour descriptions of different moral affective values in the Affective Learning Session. In this procedure, participants viewed each face and its corresponding behaviour description presented simultaneously in pairs, with the facial stimulus above the description sentence. Average-looking faces (half female, mean rating = 4.07) were used in order to reduce undesired influence of variation in physical attractiveness across the facial stimuli. The face-sentence pair was displayed for 5s with a 300ms blank interval between trials (see Fig. 3). Each pair was presented four times, during which participants were required to imagine the currently viewed person performing the behaviour described in the corresponding sentence and to make explicit moral judgments about the behaviour. They were instructed to press “A” for a virtuous act (e.g. helped an old lady cross the road), “L” for an immoral act (e.g. tortured a cat) and Space Bar for a neutral act (e.g. walking in the street). This moral judgment task was designed to reinforce the acquired connection between the character and their moral affective value. To further control for possible physical disparities in the stimuli, we created three counterbalanced versions of the task so that each face was associated with each moral affective value once. Each participant was assigned one version of the task. After learning the character-behaviour pairs, participants were given a memory test in which they were presented with the face photos they viewed earlier and were asked to recall and report the behaviour description associated with each character. Participants who failed to demonstrate 80% or above accuracy were required to repeat the learning process until this criterion was met. Once they achieved at least 80% accuracy, they proceeded to the binocular rivalry task session described previously. As in Experiment 1, dominance duration was recorded as the dependent variable in a total of 72 trials for each participant.

In Experiment 3, 31 participants (12 males) went through the same Affective Learning Session and Binocular Rivalry Task Session as in Experiment 2. We used the same stimuli and learning criterion as in Experiment 2. However, because we only investigated the effect of positive moral affective values in comparison to neutral values, we excluded the six descriptions of immoral behaviours and their corresponding characters. Because of this, only two counterbalanced task versions were created. Data were collected from a total of 48 binocular rivalry trials for each participant.

**Table A.** Summary of Descriptive Statistics of Dominance Duration in Experiment 1 (Mean ± Std.)

| Physical Attractiveness | Male Participants | | Female Participants | |
| --- | --- | --- | --- | --- |
|  | Male Faces | Female Faces | Male Faces | Female Faces |
| Beautiful | 4059(1197) | 4226(1347) | 3404(1328) | 4025(1813) |
| Average | 3424(1464) | 3559(1326) | 3285(1497) | 3301(1509) |
| Unattractive | 3051(1284) | 3151(1542) | 2825(1385) | 3041(1438) |

**Table B.** Summary of Descriptive Statistics of Dominance Duration in Experiment 2 (Mean ± Std.)

| Morality Level | Male Participants | | Female Participants | |
| --- | --- | --- | --- | --- |
|  | Male Faces | Female Faces | Male Faces | Female Faces |
| Virtuous | 3599(654) | 3871(860) | 2697(940) | 3049(922) |
| Neutral | 3471(877) | 4052(723) | 2724(778) | 3085(957) |
| Immoral | 3753(1070) | 4079(615) | 3003(636) | 3429(975) |

**Table C.** Summary of Descriptive Statistics of Dominance Duration in Experiment 3 (Mean ± Std)

| Morality Level | Dominance Duration |
| --- | --- |
| Virtuous | 3825(1344) |
| Neutral | 3384(1234) |
